# Campbell Title Registrations to Date—September 2025, and Discontinued Protocols

**DOI:** 10.1002/cl2.70085

**Published:** 2025-12-01

Details of new titles for systematic reviews or evidence and gap maps that have been accepted by the Editor of a Campbell Coordinating Group are published in each issue of the journal. If you would like to receive a copy of the approved title registration form, please send an email to the Managing Editor of the relevant Coordinating Group.

A list of discontinued protocols appears below these new titles. If you are interested to continue a project, please get in touch with the Managing Editor of the relevant Coordinating Group or email info@campbellcollaboration.org.

**Ageing**

Mapping Financial Literacy Programs for the Ageing Population: Protocol for a Scoping Review populations

Carina Sofia Teixeira Fernandes, Isabel Silva, Inês Gomes

2 September 2025

Peer‐supported intervention to improve diabetes related quality of life and diabetes management in the elderly with type 2 diabetes: A systematic review

Segufta Dilshad, Cheong Theng, Sazlina Ghazali, Lim Ying

29 September 2025

**Business and Management**

Does Nonfinancial Information Disclosure Predict Stock Market Outcomes?–A systematic review

Hongli Shang, Yi Sheng, Guoling Qiang, Wenjie Zhou, Yufeng Wen, Yongjie Feng, Weijie Lin

4 November 2025

Effects of Organizational Transparency Interventions on Trust in AI‐Assisted Decision‐Making and Human Decision‐Makers: A Systematic Review of Enabling Mechanisms

Rachel Hor, Michael Zhang

10 November 2025

**Climate Solutions**

Climate Shocks and Intimate Partner Violence in Low‐ and Lower Middle‐Income Countries: A Protocol for a Scoping Review

Kelly Murray, Savannah Badt, Shalean Collins Kapur, Stella Neema, Anita Raj, Martha Silva, Monica Swahn, Anastasia Gage

24 October 2025

Coastal land reclamation and climate risk: an evidence and gap map

Jan Petzold, Charlotta Mirbach, Robert Nicholls

27 October 2025

**Education**

Interventions to Mitigate Gender Bias in Healthcare Education and Clinical Training: A Scoping Review

Laura Rodríguez‐González, Margarita Bernales

6 October 2025

**International Development**

Tax free pad versus taxed pad policy for menstrual hygiene management globally: A systematic Review

Ndum Ayeah, Nian Yuh, Damaris Ntam, Constance Ayongho, Ernest Wung, Patrick Mbah

22 September 2025

**Knowledge Translation and Implementation**

Barriers and facilitators to the implementation of the Diabetes Prevention Program (DPP) in health settings: Protocol for a mixed methods systematic review

Miquel Colom‐Rosselló, Manuela Abbate, Laura Capitan‐Moyano, Elena Pastor‐Ramon, Maria Fernandez, Aina Yañez, Miquel Bennasar‐Veny

18 September 2025

How do General Practitioners share knowledge and seek advice regarding paediatric care? Mixed methods systematic review

Tina Vickery, Georgia Fisher, Lisa Pagano, Darran Foo, Jeffrey Braithwaite

18 September 2025

**Social Welfare**

Mapping Psychosocial Treatment Outcomes for Substance Use Disorders: A Scoping Review

Nicola Worcman, Silvana Proenca Marchetti, Lucas Oliveira Maia, Luís Fernando Tófoli

4 September 2025

Measures of parenting constructs utilised with Indigenous caregivers in Canada, Australia, Aotearoa and the United States: A scoping review

Alexandra Gregory, Kim Caudwell, Leeann Mick‐Ramsamy, Sara Filoche, Emily Armstrong, Gary Robinson, Kayli Wild

6 October 2025

The impact of phubbing in official contexts: A systematic review and meta‐analysis of workplace and academic settings

Fatemeh Shahrajabian, Jafar Hasani, Sara Shirmardi

6 October 2025

**Discontinued protocols**

If you are interested to continue one of the projects below, please get in touch with the Managing Editor of the relevant Coordinating Group or email info@campbellcollaboration.org.

**Crime and Justice**

https://onlinelibrary.wiley.com/doi/10.1002/CL2.85

PROTOCOL: Mandatory arrest for misdemeanor domestic violence effects on repeat offending

Barak Ariel, Lawrence W. Sherman

**Education**

https://onlinelibrary.wiley.com/doi/10.1002/CL2.215

Protocol for a systematic review: Interventions to Improve Mathematics Achievement in Primary School‐Aged Children: A Systematic Review

Victoria Simms, Camilla Gilmore, Seaneen Sloan, Clare McKeaveney

https://onlinelibrary.wiley.com/doi/10.1002/cl2.1011

PROTOCOL: Protocol for a systematic review: Interschool Collaborations for Improving Educational and Social Outcomes for Children and Young People: A Systematic Review

Paul Connolly, Jennifer Hanratty, Joanne Hughes, Christopher Chapman, Danielle Blaylock

https://onlinelibrary.wiley.com/doi/10.1002/CL2.145

PROTOCOL: Practices and Program Components for Enhancing Prosocial Behavior in Children and Youth: A Systematic Review

Asha L. Spivak, Mark W. Lipsey, Dale C. Farran, Joshua R. Polanin

https://onlinelibrary.wiley.com/doi/10.1002/CL2.185

PROTOCOL: The Direct and Indirect Effects of School‐Based Executive Function Interventions on Children and Adolescents’ Executive Function, Academic, Social‐Emotional, and Behavioral Outcomes: A Systematic Review

Saiying Steenebergen‐Hu, Paula Olszewski‐Kubilius, Eric Calvert

https://onlinelibrary.wiley.com/doi/10.1002/CL2.163

PROTOCOL: Provision of Information and Communications Technology (ICT) for Improving Academic Achievement and School Engagement in Students Aged 4‐18

Kristin Liabo, Laurenz Langer, Antonia Simon, Kathy‐ann Daniel‐Gittens, Alex Elwick, Janice Tripney

https://onlinelibrary.wiley.com/doi/10.1002/CL2.210

PROTOCOL: Universal school‐based programmes for improving social and emotional outcomes in children aged 3–11 years

Paul Connolly, Sarah Miller, Jennifer Hanratty, Jennifer Roberts, Seaneen Sloan

https://onlinelibrary.wiley.com/doi/10.1002/CL2.110

PROTOCOL: Education support services for improving school engagement and academic performance of children and adolescents with a chronic health condition

Michelle A. Tollit, Susan M. Sawyer, Savithiri Ratnapalan, Tony Barnett

https://onlinelibrary.wiley.com/doi/10.1002/CL2.197

PROTOCOL: Electronic mentoring to promote positive youth outcomes for young people under 25: a systematic review

Robyn M. O'Connor, David L. DuBois, Lucy Bowes

https://onlinelibrary.wiley.com/doi/10.1002/CL2.166

PROTOCOL: Merit Pay Programs for Improving Teacher Retention, Teacher Satisfaction, and Student Achievement in Primary and Secondary Education: A Systematic Review

Gary Ritter, Julie Trivitt, Leesa Foreman, Corey DeAngelis, George Denny

**International Development**

https://onlinelibrary.wiley.com/doi/10.1002/cl2.1122

PROTOCOL: Development evaluations in India 2000–2018: A country impact evaluation map

Ashrita Saran, Eti Rajwar, Bhumika T.V., Divya S. Patil, Howard White

https://onlinelibrary.wiley.com/doi/10.1002/cl2.1186

PROTOCOL: The association between whole‐grain dietary intake and noncommunicable diseases: A systematic review and meta‐analysis

Wasim A. Iqbal, Gavin B. Stewart, Abigail Smith, Linda Errington, Chris J. Seal

https://onlinelibrary.wiley.com/doi/10.1002/CL2.84

PROTOCOL: Nutrition interventions and programs for reducing mortality and morbidity in pregnant and lactating women and women of reproductive age: a systematic review

Philippa Middleton, Caroline Crowther, Tanya Bubner, Vicki Flenady, Zulfiqar Bhutta, Tran son Trach, Zohra Lassi

https://onlinelibrary.wiley.com/doi/10.1002/CL2.138

PROTOCOL: Improving maternal, newborn and women's reproductive health in crisis settings

Primus Che Chi, Henrik Urdal, Odidika UJ Umeora, Johanne Sundby, Paul Spiegel, Declan Devane

https://onlinelibrary.wiley.com/doi/10.1002/CL2.183

PROTOCOL: The impacts of interventions for female economic empowerment at the community level on human development

Marcela Ibanez, Sarah Khan, Anna Minasyan, Soham Sahoo, Pooja Balasubramanian

https://onlinelibrary.wiley.com/doi/10.1002/CL2.154

PROTOCOL: Peer‐based interventions for reducing morbidity and mortality in HIV‐positive women

J Petkovic, M Doull, AM O'Connor, J Aweya, M Yoganathan, V Welch, GA Wells, P Tugwell

https://onlinelibrary.wiley.com/doi/10.1002/CL2.188

PROTOCOL: Community‐led total sanitation in rural areas of low‐ and middle‐income countries: a systematic review of evidence on effects and influencing factors

Josef Novotný, Jiří Hasman, Martin Lepič, Vít Bořil

https://onlinelibrary.wiley.com/doi/10.1002/CL2.140

PROTOCOL: Access to electricity for improving health, education and welfare in low‐ and middle‐income countries

Kavita Mathur, Sandy Oliver, Janice Tripney

https://onlinelibrary.wiley.com/doi/10.1002/CL2.157

PROTOCOL: Saving promotion interventions for improving saving behaviour and reducing poverty in low‐ and middle‐income countries

Janina Isabel Steinert, Ani Movsisyan, Juliane Zenker, Ute Filipiak, Yulia Shenderovich

https://onlinelibrary.wiley.com/doi/10.1002/CL2.172

PROTOCOL: The effect of interventions for women's empowerment on children's health and education

Sebastian Vollmer, Sarah Khan, Le Thi Ngoc Tu, Atika Pasha, Soham Sahoo

https://onlinelibrary.wiley.com/doi/10.1002/CL2.200

PROTOCOL: The effects of road infrastructure, and transport and logistics services interventions on women's participation in informal and formal labour markets in low‐ and middle‐income countries

Manisha Gupta, Souvik Bandyopadhyay, Meerambika Mahapatro, Shreya Jha

https://onlinelibrary.wiley.com/doi/10.1002/cl2.1135

PROTOCOL: Water, sanitation and hygiene for reducing childhood mortality in low‐ and middle‐income countries

Hugh Sharma Waddington, Sandy Cairncross

**Social Welfare**

https://onlinelibrary.wiley.com/doi/10.1002/cl2.1056

PROTOCOL: Psychosocial interventions for preventing PTSD in children exposed to war and conflict‐related violence: A Systematic Review

Jennifer Hanratty,

Laura Neeson, Tania Bosqui, Michael Duffy, Laura Dunne, Paul Connolly

https://onlinelibrary.wiley.com/doi/10.1002/cl2.1259

PROTOCOL: Effects of virtual reality exposure therapy versus in vivo exposure in treating social anxiety disorder in adults: A systematic review and meta‐analysis

Martin Polak, Norbert Tanzer, Per Carlbring

https://onlinelibrary.wiley.com/doi/10.1002/CL2.109

PROTOCOL: The therapeutic alliance and psychotherapy outcomes for young adults aged 18 to 34

Stacy J. Green, Julia H. Littell, Karianne Hammerstrøm, Emily Tanner‐Smith, Jessica Schaffner Wilen

https://onlinelibrary.wiley.com/doi/10.1002/CL2.56

PROTOCOL: Cognitive‐behavioural therapy for parents who have physically abused their children

Mogens N. Christoffersen, Jacqueline Corcoran, Diane DePanfilis, Claire Daining.

